# Persistence and Sexual Transmission of Filoviruses

**DOI:** 10.3390/v10120683

**Published:** 2018-12-02

**Authors:** Brayden G. Schindell, Andrew L. Webb, Jason Kindrachuk

**Affiliations:** Laboratory of Emerging and Re-Emerging Viruses, Department of Medical Microbiology and Infectious Diseases, University of Manitoba, Winnipeg, MB R3E 0J9, Canada; schindeb@myumanitoba.ca (B.G.S.); webba2@myumanitoba.ca (A.L.W.)

**Keywords:** Ebola virus, persistence, testis, filovirus, emerging virus, outbreak, sexual transmission, public health, blood-testis barrier

## Abstract

There is an increasing frequency of reports regarding the persistence of the Ebola virus (EBOV) in Ebola virus disease (EVD) survivors. During the 2014–2016 West African EVD epidemic, sporadic transmission events resulted in the initiation of new chains of human-to-human transmission. Multiple reports strongly suggest that these re-emergences were linked to persistent EBOV infections and included sexual transmission from EVD survivors. Asymptomatic infection and long-term viral persistence in EVD survivors could result in incidental introductions of the Ebola virus in new geographic regions and raise important national and local public health concerns. Alarmingly, although the persistence of filoviruses and their potential for sexual transmission have been documented since the emergence of such viruses in 1967, there is limited knowledge regarding the events that result in filovirus transmission to, and persistence within, the male reproductive tract. Asymptomatic infection and long-term viral persistence in male EVD survivors could lead to incidental transfer of EBOV to new geographic regions, thereby generating widespread outbreaks that constitute a significant threat to national and global public health. Here, we review filovirus testicular persistence and discuss the current state of knowledge regarding the rates of persistence in male survivors, and mechanisms underlying reproductive tract localization and sexual transmission.

## 1. Introduction

### 1.1. Introduction to Filoviruses

The family *Filoviridae* (order *Mononegavirales*) includes three genera of single-stranded, negative-sense RNA viruses that are enveloped, filamentous, and non-segmented [1,2]. There are six species of *Ebolavirus,* one species of *Marburgvirus*, and one species of *Cuevavirus* [3,4]. Species within the genus *Ebolavirus* include: *Bundibugyo ebolavirus* (BDBV), *Zaire ebolavirus* (EBOV), *Reston ebolavirus* (RESTV), *Sudan ebolavirus* (SUDV), *Taï Forest ebolavirus* (TAFV), and the recently discovered *Bombali ebolavirus* (BOMV) [5]. The genus *Marburgvirus* comprises a single species, *Marburg marburgvirus*, that encompasses the Marburg virus (MARV) and the Ravn virus (RAVV). The genus *Cuevavirus* contains one species, *Lloviu cuevavirus* (LLOV), which has yet to be isolated [6].

Marburg virus disease (MVD) was first recognized in 1967 in West Germany and Yugoslavia following a number of infections transmitted from African green monkeys imported from Uganda, and Ebola virus disease (EVD) was recognized in 1976 when EBOV and SUDV emerged in the Democratic Republic of the Congo (formerly Zaire) and South Sudan (formerly southern Sudan), respectively [7]. Ebolaviuses and marburgviruses are etiological agents with high infection and mortality rates for humans and non-human primates [8,9,10,11,12]. MVD case fatality rates have ranged from 24–88% [13]. The mean case fatality rates of EBOV, SUDV, and BDBV in human outbreaks have ranged from 30–50% [14]. TAFV has been associated with a single non-fatal human infection and RESTV results in asymptomatic infections in humans [2,15,16]. RESTV is the only *Ebolavirus* species to have emerged outside of Africa and has been associated with multiple epizootics in captive Philippine macaques [17,18].

The number and magnitude of EVD and MVD outbreaks have increased over time, likely due in part to greater interaction between humans and intermediate or reservoir host populations [19]. Historically, EVD and MVD outbreaks were limited to hundreds of cases in isolated rural locations [19,20]. In comparison, the recent West African EVD epidemic lasted two years and resulted in 28,652 cases and 11,325 fatalities across three countries (including suspect, probable, and confirmed cases) [19]. After the West African epidemic subsided, surveillance of EVD survivors revealed that EBOV persists in the male reproductive tract for extended periods of time in the absence of symptoms [21,22,23,24]. While persistence had been noted in cases studies in previous outbreaks of EVD and MVD, the scale of the West African epidemic led to targeted surveillance of survivors in numbers suitable for statistical analysis [25,26,27,28]. A recent modeling study demonstrated that filovirus testicular persistence likely occurs in a high proportion of male disease survivors [29]. The unprecedented scale of the West African EVD epidemic and the risk that persistent filovirus infections could result in the transmission of EBOV to new geographic regions constitutes a significant threat to global public health.

### 1.2. Filovirus Pathophysiology

The recent review by Baseler et al. summarizes the current state of knowledge regarding EVD pathophysiology in humans following observations from the West African EVD epidemic, and additional reviews are available elsewhere [30,31,32,33]. We will briefly discuss the pathophysiological features that have been associated with filovirus infections. Transmission likely occurs through mucosal surfaces, skin abrasions, or parenteral introduction following direct contact with symptomatic patients or deceased patients [34,35,36,37]. The mean incubation times associated with EVD and MVD range from 3–12 days and 5–9 days, respectively, and median survival is nine days following the onset of clinical symptoms [20]. EVD symptoms typically begin prior to detectable viremia and are often accompanied by fever, malaise, fatigue, muscle weakness, and myalgia [28,38,39,40,41]. Filovirus infections are difficult to diagnose during the early stages of disease because the associated symptoms are common for many infectious agents present in the same geographic region (e.g., malaria, respiratory viruses, arboviruses, leptospirosis, and enteric bacterial pathogens) [33]. Thrombocytopenia, leukocytosis, elevated liver enzymes, and coagulation defects (including disseminated intravascular coagulation, prolonged prothrombin time, and partial thromboplastin time) are commonly associated with clinical illness [28,38,39,41,42,43]. Patient data from fatal and non-fatal EVD cases suggests that while the initial onset and increase in viremia are similar in both groups, higher peak viremia and slower rates of viral decline are often associated with fatal disease [44,45,46,47]. The development of severe hypotension and shock also appear to differentiate fatal from non-fatal diseases [28,36,37,48,49]. Although ebolaviruses and marburgviruses have traditionally been considered as hemorrhagic fever viruses, hemorrhage is found in less than half of EVD and MVD patients [31]. Hemorrhagic manifestations are predominantly localized to mucosa and conjunctivae tissues and may be associated with persistent bleeding from venipuncture sites [28,39,48,50,51,52,53]. Petechiae and mucosal hemorrhage result in altered fluid distribution, hypotension, and aberrant coagulopathy during peak illness [54,55,56,57].

## 2. Filovirus Pathogenesis

### 2.1. Filovirus Cell Tropism 

In the past, the spontaneous emergence and inaccessible geographic locations of filovirus outbreaks hindered direct studies of viral pathogenesis in humans [37,48]. Cultural considerations and biosafety concerns (as well as jurisdictional difficulties) have impeded biological sample collection during filovirus outbreaks [58,59]. As a result, the current understanding of the host and viral molecular factors involved in filovirus pathogenesis has been based primarily on animal model and in vitro studies as well as limited biological samples collected from infected and deceased patients [60,61,62]. Complete reviews of the current state of understanding of filovirus cellular tropism and pathogenesis are available elsewhere [63,64,65]. Briefly, filovirus pathogenesis begins with targeted infection of the immune system, which causes widespread immune dysregulation [66,67]. Widespread immune dysregulation caused by conflicting pro-inflammatory and anti-inflammatory regulators allows filoviruses to infect a range of susceptible tissues, including the spleen, kidneys, liver, lungs, and gastrointestinal tract [63]. As the infection progresses, the barrier function of endothelial cells is impaired [65]. At some point during the late stages of infection, filoviruses infiltrate sensitive tissues such as the eye, brain, and reproductive tract [62,63,68]. Aside from supportive medical care, the differentiation between fatal and non-fatal diseases largely depends on the poorly understood interaction between filoviruses and the host immune system.

### 2.2. Immune Response 

Immune responses consist of innate and adaptive cells that detect pathogens, recruit additional immune cells to the site of infection, and initiate antiviral activity [69]. MVD and EVD are notable for delayed immune responses and widespread cellular necrosis in the absence of observable inflammation [70,71,72]. These delayed responses are attributed to the viral-mediated inhibition of inflammatory immune responses and interferon (IFN) signalling pathways [73]. Normally, invading viruses are detected by the innate immune system and a signalling cascade culminates in the upregulation of antiviral genes in infected and neighboring cells [74]. For EBOV, proteins VP35 and VP24 inhibit the induction of type 1 IFN responses [75,76]. In contrast, type 1 IFN antagonism is directed by VP40 in MARV [73]. In addition, infected dendritic cells and macrophages release negative immune regulators such as interleukin 10 (IL-10) and programmed death-ligand 1 to suppress natural killer (NK) and T lymphocyte cells, which in turn prevents a pro-inflammatory response, the destruction of infected cells, and/or the generation of memory cells [74]. Death in late-stage fatal illness may be related to the excessive release of inflammatory factors (i.e., cytokine storm) [54,77]. 

### 2.3. Immune-Privileged Niches

To minimize damage to host cells, pro-inflammatory immune responses are followed by a release of anti-inflammatory factors. Tissues at greater risk of damage have modified local immune responses that heavily suppress pro-inflammatory responses [78,79]. These immune-privileged niches (IPN) include the eye, brain, and reproductive tract. Tolerance also appears to contribute to immune privilege in additional organs, including: (i) the suppression of T cells by sinusoidal endothelial cells, dendritic cells, Kupffer cells, and hepatocytes in the liver; (ii) the maintenance of immune homeostasis in the lung by regulatory T cells and natural killer T cell subsets; and (iii) immune homeostasis in the gut by gut-associated lymphoid tissue [78]. The current understanding of the interaction between viruses and host immune response in these niches varies by tissue [78,80]. Immune privilege can include physical segregation of sensitive tissues away from immune cells, reduced cytokine responsiveness, and increased cytokine regulation [81,82,83,84]. Immune cells are prevented from interacting with cells in the testes and the brain by means of blood-tissue barriers. In the testes, interstitial cells proximal to the blood-testis barrier (BTB) maintain a degree of immune privilege; macrophages are less active and dendritic cells are immature at the BTB. Finally, cells within IPNs often possess a level of anti-inflammatory immune regulation. While immune privilege is necessary to protect sensitive cells from damage due to inflammation, these sites are also prime targets for infection [79].

### 2.4. Filovirus Persistence in Immune-Privileged Niches

Pathogen persistence is the sum result of positive and negative immune regulation resulting in an immune-tolerant environment that can mean a lack of visible symptoms [85]. Thus, if immune homeostasis becomes compromised, it can lead to viral elimination or acute phase relapse [86,87]. There is evidence that host cell activity changes based on viral activity. For example, CD8 T cells that interact with major histocompatibility complex molecules differ markedly between acute and chronic infections [88,89,90]. Despite the fact that filoviruses induce apoptosis in T cell lymphocytes, CD4 T cells aid in the struggle during persistent infections [91]. The fragile balance between host and viral factors is evidenced by experiments in which the semi-functional type 1 IFN system is suppressed exogenously, which causes persistently infected cells to return to acute phase-like replication [92]. Host resistance to persistent viral infection is focused on preventing viral protein translation, packaging and release compared to viral replication and transcription [92]. A host strategy of defense may work to mitigate both inflammation and infection of new cells while the persistent infection burns itself out [87]. To this end, the establishment of persistent filovirus infections late in the acute infection cycle could be potentiated by an increasing ratio of truncated viral genomes due to error-prone viral replication machinery that results in the generation of replication-incompetent virions that are able to bind to and enter cells [93]. Referred to as defective interfering particles (DIP), these truncated genomes compete for cellular machinery and thus increasingly reduce the replication rate of replication-competent virions [94]. The suitability of immune-privileged sites for viral persistence is evident in the continued detection of pathogenic material in these sites long after they are no longer detectable in blood and most of the body. Filovirus material persisting in immune-privileged sites has been shown to decrease steadily over time until it is no longer detected, but it can take months to years for persistent EBOV to be cleared. This eventual clearance may indicate that the balance of power during persistence is in the host’s favour.

**Figure 1:**
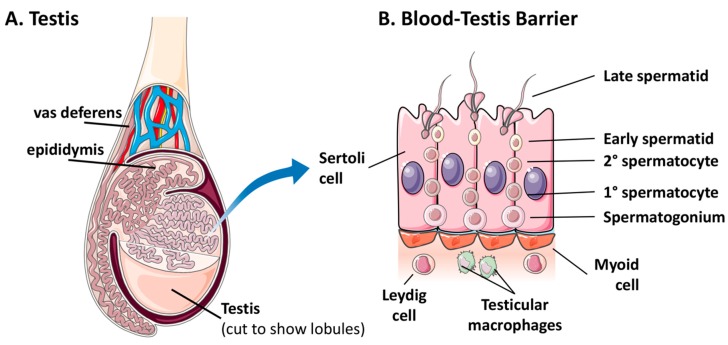
Localization of the blood-testis barrier within the male reproductive tract. (**A**) Localization of the seminiferous tubule within the testis; (**B**) Cell composition and structure of the blood-testis barrier and contribution of surrounding cells to the barrier function and homeostasis. Tissue and cell images were derived and/or modified from Servier Medical Art under a Creative Commons Attribution 3.0 Unported License.

There is a paucity of information regarding the persistence of viral pathogens in immune-privileged sites. Infection of immune-privileged sites is likely a targeted strategy of persistence, in which acute infections shift to a persistent nature in response to an increasing host immune response [87]. Viral material has been detected in the ocular fluid and semen of human survivors of EVD for months or years post-recovery, which indicates that viral persistence is likely occurring in the eye and testes, respectively [23,29,68,95,96,97,98]. In addition, EBOV persistence in the central nervous system has resulted in relapse and acute disease in an EVD survivor [99]. While symptoms of persistent EBOV infections of ocular and brain tissue have been reported, along with sequelae attributed to acute and/or persistent infections in these compartments, persistent testicular infections appear to be asymptomatic [68]. Perhaps as a result, there is a scarcity of data regarding the host and viral molecular factors that contribute to asymptomatic EBOV persistence in the male reproductive tract. EBOV persistence in the testes in the absence of symptoms may be attributed to the unique nature of the BTB [68]. The BTB separates developing germ cells from the blood stream, and therefore from circulating adaptive immune cells [79]. Physical separation of germ cells is accomplished by a layer of myoid cells over tightly joined Sertoli cells (Figure 1). In addition, several cells that form or interact with the BTB also appear to possess immunosuppressive properties [81]. Considering that EBOV infection of the testes facilitates both the persistence and transmission of this virus, it is critical that the molecular dynamics of host and virus interaction at the BTB be elucidated. 

## 3. Male Testicular Persistence

Although filoviruses emerged as high-consequence human pathogens over 40 years ago, there is growing concern regarding viral testicular persistence as a novel transmission mechanism. First reported in 1968 following the MVD outbreak, filovirus testicular persistence has grown from an anomaly to a major public health concern based on observations from the West African EVD epidemic [100,101]. Recently, there has been an increasing focus on the mechanisms underlying such persistence. Longitudinal studies have focused on the length of viral persistence and viral evolution within the male reproductive tract [29,68,98,102]. 

Research on EBOV persistence has been ongoing to varying degrees since the 1995 EVD outbreak in Kikwit, Democratic Republic of Congo [26,103]. Although animal models of filovirus infections are routinely used for viral pathogenesis and therapeutic efficacy studies, only a few investigations have examined viral persistence. As a result, current knowledge regarding filovirus persistence has been gleaned from clinical case reports. 

### 3.1. In Vivo Models of Study

To date, there have been sporadic studies examining filovirus testicular persistence in vivo. These investigations date back to the initial MVD outbreak, when guinea pigs were used to detect the virus in patient samples and for subsequent viral propagation and isolation [100]. Examinations to detect infectious virus in samples that have tested positive for viral genomic material continues to this day in small animals [27,29,104]. The use of animals to model viral pathogenesis also began fairly early following the discovery of filoviruses with increased emphasis on EBOV infections [105,106]. Although rodents are routinely used to study EBOV pathogenesis, the virus must be adapted through serial passage in order to cause symptomatic infection [106,107,108,109,110,111]. Small animal models of filovirus infections have facilitated efficacy testing for antivirals and vaccines, including those utilized in recent EVD outbreaks [107,112,113,114]. 

The use of animal models to examine EBOV persistence has increased following the West African EVD epidemic. Bird et al. recently demonstrated that wild-type EBOV infection in humanized mice results in symptomatic disease with fatality rates similar to those seen in humans [60]. In addition, EBOV was detectable in the testes of mice that survived infection. Histopathological analysis of guinea pigs infected with guinea pig-adapted EBOV demonstrated that the virus infects the liver and spleen, resulting in widespread necrosis [106,115]. The presence of EBOV and tissue necrosis were also noted in the reproductive tracts of female (three animals) and male (one animal) guinea pigs [115]. 

Filovirus reproductive tract persistence has been examined in non-human primates. A recent investigation found that asymptomatic testicular persistence occurred in 9.82% of non-fatal EBOV infections in rhesus macaques [116]. The virus was identified in the epididymis but absent in the testes; however, it should be noted that this was a single animal and further investigation is thus required [116]. An additional investigation demonstrated EBOV localization within the reproductive tissues of male and female rhesus and cynomolgus macaques with viral deposition occurring during the acute phase of infection [117]. Immunohistochemistry demonstrated that EBOV was found in the ovary and uterus of the females and within the testes, prostate gland, seminal vesicle, and epididymis of males [117]. A recent study by the same authors analyzed historical tissues from animals that survived MARV infection and represents the most in-depth study of filovirus testicular persistence performed thus far [118]. Viral testicular persistence was identified in 22 of 73 animals with BTB disruption noted in persistently infected animals accompanied by the infiltration of leukocytes, macrophages, T cells, and B cells in the seminiferous tubules and depletion of germ cells. Testicular transmission occurred following peak illness, with Sertoli cells being the primary target for the virus [118]. While there have been increasingly detailed mechanistic and pathological studies of filovirus reproductive tract persistence, many questions are yet to be answered.

### 3.2. Treatment and Immune Barriers

There are many post-exposure treatments under investigation for EVD. Recently, ZMapp, an antibody cocktail, and Favipiravir, a small molecule therapeutic, were deployed for patient treatment during the West African epidemic [113,119,120,121]. A longitudinal persistence study included several patients treated acutely with Favipiravir and did not demonstrate any efficacy for the prevention of EBOV persistence in male survivors [29]. Although the rVSV-EBOV vaccine has been used for healthcare workers during recent outbreaks with no adverse reactions [114,119,122], concerns remain regarding the viral escape to, and persistence within, immune-privileged niches [29]. 

Remdesivir (GS-5734), an adenosine nucleoside analogue that has demonstrated efficacy in EBOV-infected non-human primates, has the potential to enter immune-privileged sites [112,123]. Remdesivir is currently being examined as a potential preventative of EBOV testicular persistence in a phase 2 clinical trial consisting of EVD survivors [123,124]. In this study, part of PREVAIL IV, antiviral activity and safety are being assessed in male survivors with testicular persistence as there are currently no treatments that can clear EBOV from the reproductive tract [124]. Continued investigations of treatment strategies that target filovirus testicular persistence will help reduce the potential for the initiation of new chains of disease transmission or viral transmission to new geographic locations due to persistent infections within disease survivors.

### 3.3. Clinical Case Reports of Persistence

Numerous case reports have been published on filovirus infections in human patients since the emergence of filoviruses [125]. These investigations have been critical in helping to guide clinically-relevant filovirus research and have contributed to the current state of knowledge of MVD and EVD. The clinical pathology and symptoms observed for MVD and EVD have facilitated the development of clinical profiles for these diseases. 

#### 3.3.1. Testicular Persistence-Associated Pathology (1967–1990)

Pathological investigations of deceased patients were performed on five of the seven individuals that succumbed to infection during the 1967 MVD outbreak in Marburg, Germany [125]. Eighty percent of the male fatalities (4/5) had signs of necrosis or fluid accumulation (inflammation) within the testes at autopsy, suggesting viral-mediated pathology [125]. Swelling was reported in 5/16 male patients in Marburg and 4/5 in Frankfurt reported some form of scrotal abnormality (pain and/or orchitis) [100,126,127]. One of the patients in Marburg did not have testicular pain and swelling until over 36 days following the initial symptom onset, falling well outside of the acute illness period [126]. Orchitis was also observed in a laboratory-acquired MARV infection in Russia in 1990 [104]. Testicular swelling of four times the normal size was observed 52 days after hospital admission for a duration of 4–5 days [104]. These observations of testicular pain and orchitis in MVD survivors are important to note as no such reports have been made in any case reports or cohort studies of EVD. Further suggesting a difference between EVD and MVD, a recent pathology study of testicular tissue from a fatal EVD patient found no inflammation in the testicular tissue with viral antigen localized to the seminiferous tissue and interstitial cells [63].

#### 3.3.2. Viral Isolation (1967–2000)

In early case reports, EBOV and MARV were identified in sperm cells or seminal fluid similar to that observed during the recent West African EVD epidemic (Table 1) [25,26,27,62,100,104,128]. Viral presence in sperm was first noted during the 1967 MVD outbreak, with semen samples testing positive at 12 weeks post-infection [100]. EBOV was subsequently cultured from semen in 1976, 18 days after patient admission to hospital and was treated as a harmless artifact and not a concern for the hospital staff [27]. Seminal fluid samples were positive up to 61 days following admission [27]. MARV was recovered from the seminal fluid of a patient two months following disease recovery in Kenya in 1980 [25], as well as 84 days after hospital admission following an infection in Siberia, Russia [104]. During the 1995 EVD outbreak in Kikwit, Democratic Republic of Congo, 8 patients were identified with persistent testicular infections [26]. Viral genomic material was detected in seminal fluid for up to 101 days, with infectious virus isolated from a single seminal fluid sample 82 days post-onset. SUDV was detected in a semen sample from a patient during the 2000 outbreak in Uganda, where virus was detected 40 days post-onset by RT-qPCR and culture [128]. 

Collectively, decades of clinical data have demonstrated that filovirus family members likely have different pathological presentations associated with testicular persistence [100,104]. Despite a continually increasing number of filovirus outbreaks since the 2000s, few reports have focused on the semen/testicular localization of these viruses. 

#### 3.3.3. West African Outbreak (2014–2016)

Several studies conducted during the West African EVD epidemic centering on testicular persistence and sexual transmissions with advanced methodologies have provided important insights into this biological phenomenon (Table 2). 

One of the first reported cases of testicular persistence during the epidemic is thought to have resulted in persistence for 199 days post-EVD onset [130]. During an investigation into fatal EVD in an infant in August 2015, it was discovered that the father of the infant tested positive for viral RNA within his semen [129]. However, clinical signs of disease were not seen in either the mother or father of the child prior to the infection of the infant [129]. The total length of the father’s persistent EBOV infection is unknown though the virus was shown to have persisted for 30 days during the course of the epidemiological investigation [129]. An epidemiological survey of cases in Sierra Leone identified a chain of transmission in Kambia stemming from a persistently infected male with matching EBOV genomes in his initial acute blood sample (July 2015) and his seminal sample obtained 52 days following discharge from an Ebola treatment center (September 2015) [98]. No changes in viral genome sequence were found between the acute sample and the seminal sample, suggesting that the virus likely underwent a slowed rate of replication during persistence [98]. These studies demonstrate the difficulties in assessing mild or asymptomatic EVD cases that could contribute to the initiation of new chains of transmission.

A US healthcare worker infected in Sierra Leone was transported to the National Institutes of Health Special Clinical Studies Unit in Bethesda, MD, following EVD onset and recovered with the aid of advanced supportive care [68]. High viral loads (including viral genome and infectious virus) were detected in the patient’s semen. Peak viral genome copies in the semen were up to 10,000 times higher than those found in the blood at peak viremia and in the absence of viral genome diversification. EBOV has also been found in semen for far more extended periods, including 531 days post-disease onset [132]. Diallo et al. examined the evolutionary history of the virus over the 531-day period and showed that the virus was identical to that found during the acute phase of disease and demonstrated a ten-fold reduction in the evolutionary rate during testicular persistence [132]. The observations by Barnes et al. and Diallo et al. demonstrate a similar reduction in the mutation/evolution rate during persistent testicular infection [68,132]. Additional investigations of the rates and lengths of testicular persistence will aid future outbreak containment and patient treatment strategies.

### 3.4. Longitudinal Cohort Studies

There have been nine seminal/testicular persistence longitudinal cohort studies since 1995 that have followed a total of 158 persistent male survivors with seven studies conducted during the West African EVD epidemic [22,23,24,26,29,62,131,133]. Two studies examining the 1995 EVD outbreak in Kikwit, Democratic Republic of the Congo, identified eight males with persistent EBOV in their semen [26]. Viral RNA was detected up to 101 days post-disease onset and live virus cultured up to 82 days post-onset [26]. These investigations have served as benchmarks for subsequent examinations of persistence in male EVD survivors [23,62,130,133]. 

One of the first longitudinal studies was conducted on a cohort of five EVD patients following their return to the U.S. for treatment during the West African EVD epidemic [23]. Persistence ranged from 104–290 days post-EVD onset [23,26]. However, viral isolation was only reported up to 74 days post-onset [23,26]. A second longitudinal cohort study followed 8 male survivors in Guinea in 2015 [131]. This study showed wide-ranging lengths of persistence (30–276 days post-EVD onset) [23,131]. However, no culturing of infectious virus was performed on the semen samples [131].

By mid-late 2015, the seminal persistence of EBOV garnered increased attention in the scientific community as well as in mainstream media outlets due to the potential for sexual transmission [130]. From January–July 2015, semen samples from a cohort of 19 EVD survivors tested positive for EBOV in Guinea [29]. Viral genomic material was found in ten of the males >150 days post-EVD onset with persistence up to 407 days post-onset in a single survivor [29]. Infectious virus was found more than 82 days post-onset with live virus isolation >200 days post-onset in three participants, extending up to 233 days post-onset [26,29]. Sissoko et al. have provided strong evidence for the long-term testicular persistence of infectious EBOV, suggesting that patients exhibiting extended periods of persistence are at risk of sexual transmission [29]. 

Additional longitudinal studies conducted towards the end of the West African EVD epidemic have demonstrated that persistence can extend to over a year in male EVD survivors. Soka et al. examined 38 men in Liberia from 2015–2016 with persistent EBOV genomic material in their semen [22]. Persistence for >1 year was seen in 60% of the patients following their release from Ebola treatment centers. Two participants had persistent infections for over 540 days post-release with one persisting for 565 days [22]. This is of particular concern as current guidelines recommend abstinence from sexual intercourse for only nine months [134]. 

A longitudinal cohort in Guinea from late-2015 to mid-2016 identified seminal persistence of EBOV genomic material in 15 males [24]. Persistence in the patients ranged from <90 days to >540 days post-EVD onset. The largest cohort study of testicular persistence was performed in Sierra Leone in two phases from May–July 2015 and from November 2015 to May 2016, with a total of 57 persistently infected males [62]. The longest persistence of EBOV genomic material within this cohort was from 16–18 months post-release whereas the shortest period of persistence was over 3 months days post-release, longer than previously seen for minimum persistence periods. EBOV genomic material persistence has been reported for greater than 16 months post-EVD onset and up to 965 days (>2.5 years) [133]. A cohort of patients from Liberia examined from July 2016 to January 2017 had 13 participants that tested positive for EBOV genome material for >600 days post-EVD onset [133]. Although caution must exercised as some investigations have employed high-cycle threshold values (>40) for their RT-qPCR analyses, there are still several cases of EBOV persistence from 600 to >800 days post-EVD onset [133]. 

Although results have shown the potential for EBOV testicular persistence to extend over long periods of time (>16 months) in a small subset of males, it has yet to be determined if this includes infectious virus in addition to viral genome material [22,24,62,132,133]. However, Fischer et al. raise important questions regarding the potential limitations of detection (LOD) of validated EBOV diagnostics as viral RNA in semen may fall below the lower LOD of ~1000 copies/mL [133]. Furthermore, the authors also raised concerns regarding the intermittent detection of viral RNA in semen using validated diagnostic platforms and suggested that additional work must focus on whether RT-qPCR-based diagnostics are sufficient for this purpose.

### 3.5. Comparison of Longitudinal Study Findings

Rates of EBOV testicular persistence within EVD survivors are available based on multiple longitudinal cohort studies. The comparison of these data will provide important information about both the rates of persistence during the recent epidemic as well as those that can be expected in future outbreaks.

#### 3.5.1. Reported Rates of Persistence 

An analysis of persistence sought to determine the total cases of testicular persistence that resulted from the West African EVD epidemic and included data up to November 2015 from the World Health Organization (WHO) and from a cohort studied by Deen et al. [62,135]. The model predicted that 2255 men were persistently infected at the peak of the outbreak across Guinea, Liberia, and Sierra Leone, and that 73 of these would still be persistently infected by January 2016 [135]. The model was based on the assumption that the death rate was 60% and that 40% of the infected population were male. However, the data from the epidemic demonstrated that the death rate was 39.5% and the proportion of male survivors was 48.8%, suggesting that core assumptions differed from what was seen in the outbreak and that the data likely underestimated the true rates of persistence [136,137]. 

In the cohort examined by Deen et al., 100% of males had persistent testicular infections within 3 months of treatment center discharge, 62% of males remained positive from 4–6 months following discharge, 25% were positive 7–9 months after discharge, 15% were positive 10–12 months after discharge, 11% were positive 13–15 months after discharge, and 4% were positive for 16–18 months following discharge [62]. A study conducted by Subtil et al. in Guinea to help determine the predictive rates of persistence analyzed 409 semen samples from 188 male EVD survivors, with 15 participants testing positive [24]. A rate of 8.1% of participants had persistent infections with a median delay from symptom onset to participation of 310 days. Based on their data, the authors constructed a model that predicted that 31.6% of male survivors would have persistent infection up to 3 months, 13.5% up to 6 months, 2.9% up to 12 months, and 0.7% up to 18 months [24]. The authors noted that delays in study inclusion and testing resulted in reduced accuracy of the results. Nevertheless, these results will help inform future studies. An additional study in Guinea assessed EBOV persistence and extinction in 26 males (130 samples) from January–July 2015, and recruited men who had tested positive for the virus within their semen [29]. The authors demonstrated that there was a 0.58 log unit reduction in the total number of male survivors with persistent infections each month. Furthermore, the model predicted that 50% of men persisted to 115 days (90% prediction interval 72–160) post-EVD onset, 10% would still be persistent at 294 days (212–399) post-onset, and <1 patient would have been positive by July 2016. Although both models are informative, the large variations in persistence intervals between these studies should be appreciated. Based on the data from both studies, and the large number of male survivors during the West African EVD epidemic, it is likely that a large number of male EVD survivors had persistent testicular infections post-recovery. This scale of persistence leaves many opportunities for re-emergence and many challenges for control measures during EVD outbreaks. 

#### 3.5.2. Persistence Rates by Age

An investigation by Soka et al. in Liberia found that men over 40 encompassed 50% of the persistent testicular infections identified within the study but comprised only 23% of the cohort, suggesting that older men may be predisposed to increased susceptibility to persistent infections [22]. Nine percent (38) of the 429 total participants tested positive for persistent EBOV infections at least once in the study and 63% (24) of the patients were persistently infected up to 12 months post-discharge. Another study conducted in Guinea from March–October 2015 sampled 68 survivors, obtaining positive sample from eight of the men in the cohort (11.8%) [131]. The authors demonstrated that the length of EBOV testicular persistence averaged 225 days post-EVD onset in men over 40 as compared to 67.8 days in men under 40 [131]. In contrast, Sissoko et al. observed that the total length of persistent infection differed by only 12 days between men over and under 40 [22,29,131]. In addition to these studies, the two patients with the longest confirmed persistent testicular EBOV infections were men in their 40s and included persistence for 531 days and 565 days, lending support to a potential link between age and long-term persistence [132,137]. 

The studies performed to date suggest that up to 10% of male EVD survivors will have persistent testicular infections that extend beyond the nine-month abstinence window recommended by the WHO [29,62]. Data from these studies suggest that abstinence recommendations for male EVD survivors may need to be adjusted to >10 months post-EVD onset for future outbreaks, thus reducing the risk of re-emergence and new chains of transmission [29].

## 4. Filovirus Sexual Transmissions

Since the first MVD outbreak in 1967, there have been nine filovirus sexual transmission events (Table 2). Although much rarer than persistent testicular infections, similar trends are observed with the range of filovirus sexual transmission events occurring from 2–15 months post-disease onset (Table 3) [24,98,132,133]. Sexual transmissions resulting in new chains of disease transmission were observed in the West African EVD epidemic [130,138], thus highlighting the importance of the consideration of these events during future outbreaks, as will be discussed in the following sections.

### 4.1. Reported Sexual Transmissions

The first reported case of potential filovirus sexual transmission occurred during the MVD outbreak in 1967, where the wife of a male MVD patient fell ill two months following her husband’s recovery from the disease [100]. Her husband was the only MVD patient with whom she had contact, and they had resumed sexual intercourse two weeks prior to her admission to hospital [100]. Providing further evidence of sexual transmission, evidence of MARV in the husband’s semen was demonstrated by immunofluorescence for MARV antigen and positive infection of guinea pigs from semen samples [100]. A second potential sexual transmission event reported prior to the West African EVD epidemic occurred during the 1995 Kikwit EVD outbreak, though the results were inconclusive [26]. A household contact study of survivors found a female that tested weakly positive for EBOV-specific IgM antibodies and who was in contact with a persistently infected male. The female contact did not show clinical signs of infection, suggesting that she may have been asymptomatically infected. The authors postulated that she could have been infected by sexual transmission. 

The first confirmed case of filovirus sexual transmission occurred during the West African EVD epidemic in Liberia [21,130]. This transmission event was confirmed through contact tracing as the male survivor was the only known contact to have been infected with EBOV and analysis of semen confirmed a pre-existing persistent infection of 179 days prior to sexual intercourse with the patient [21,130]. Sexual transmission of EBOV was confirmed through whole viral genome analysis of the male patient’s acute blood and semen samples compared to an acute blood sample from the female patient [130]. The genomes from the three samples had a very high degree of similarity with only one nucleotide difference across the entire genome for the female and matching genomes for both acute and persistent samples from the male patient [68,130,132]. From late June to early July 2015, seven infections occurred in Needowein, Liberia, with all the sequenced viral genomes containing three mutations between them; this appeared to be a re-emergence of a virus strain from September 2014 in a different community [139]. A female who resided in both communities experienced mild symptoms and reported having a miscarriage before moving to Needowin [139]. The sequences from the two communities were one nucleotide different after nine months between the sampling dates, and suggested that a persistent survivor was the source for the flare-up [68,132,139]. However, no source of re-emergence was identified [139]. Although it was suggested that the relocated woman could have been the source of the flare-up, there was inconclusive evidence to confirm this [139]. In August 2015, a cluster of EVD cases appeared in Kambia, Sierra Leone, which clustered with an EVD patient’s acute blood sample collected from July 2015 [98]. The patient was an EVD survivor and a semen sample matched his acute blood sample. Sexual contact was confirmed between the survivor and a female from the cluster in August 2015 [98]. Samples from five additional patients also clustered with this transmission event. The patient who had sexual contact with the survivor spread her illness to four other individuals, all of whom had only three mutations in the virus as compared to the male survivor. EBOV with a genome that perfectly matched the male survivor was isolated from the daughter of the infected female; however, it is unclear how the daughter became infected though it has been suggested that this may have occurred through direct contact while providing care for ill patients [98]. In October 2015, a patient presented with EVD in Conakry, Guinea, but the genome of the virus did not match the chain of transmission [140]. It was proposed that the patient was infected by close contact with his sister, who was believed to have been asymptomatically infected by her husband, an EVD survivor, in September 2015. It is believed that the male survivor sexually transmitted EBOV to his wife 250 days after his disease onset; however, he ultimately tested negative for EBOV in his semen once he agreed to supply a sample. The similarity between the viral genomes from the infected male and the recovered male suggested an alternative chain of transmission. Although the authors note that there was no conclusive evidence for sexual transmission, they do note that the survivor’s wife was positive for EBOV IgM at the time of her brother’s infection, suggesting a potentially recent sexual transmission. In September 2015, an EVD case was reported in Bombali, Sierra Leone, where a 16-year-old girl was sick in the community for several days [141]. Interestingly there had been no reported cases of EVD from this region of Sierra Leone for five months [141,142]. No associated cases were reported and the female was confirmed to be EVD-positive post-mortem [142]. The only reports on this case were preliminary work into the epidemiology of this case and the suggestion by the WHO was that it was a case of sexual transmission [142]. Unfortunately, no follow-up has been reported. In January 2016, a deceased female tested positive for EBOV post-mortem in Sierra Leone [143]. The genome of this patient matched two genomes from November 2014, suggesting that it re-emerged from a persistent survivor [29,143]. Although a contact of the deceased female contracted EBOV from the deceased prior to death, no male survivor was traced [143]. A cluster of EVD cases occurred in N’Zerekore, Guinea, in March 2016, resulting in three community deaths and 13 confirmed cases [132]. It was determined that one of the deceased community members had sexual intercourse with a male EVD survivor that had been released from an Ebola treatment center in November 2014. The survivor abstained from sexual intercourse until September 2015 as recommended. Sexual transmission of EBOV is believed to have occurred 470 days post-disease onset in the male EVD survivor. The sequencing of an acute blood sample and semen sample (collected 504 days post-EVD onset) from the male EVD survivor was found to contain five total mutations and a reduced viral evolution rate. The survivor was confirmed as the source of the re-emergence, which was later reaffirmed with four other acute cases from the cluster matching the genome of the survivor’s semen sample [132].

### 4.2. Dynamics and Comparisons of Sexual Transmission

Although each of the described sexual transmission events is unique in its own right, there are similar trends that are important to note. As described in three reports of sexual transmission, the infected female only displayed mild symptoms or was described as asymptomatic after sexual transmission [26,139,140]. The asymptomatic nature of these cases made identification hard and confirmation even more challenging, leaving one to wonder the extent of asymptomatic sexual transmissions, as seen by Keita et al. [140]. Asymptomatic infections of the female by a persistently infected EVD survivor have been described in 3/9 reported possible or confirmed sexual transmissions to date [26,139,140]. Five of the nine reported sexual transmissions that have occurred have resulted in small outbreaks of two to 13 people, resulting in 13 total deaths [98,132,139,140,143]. Three of these five cases of sexual transmission that resulted in the initiation of small chains of transmission were initiated from females that were apparently asymptomatic [26,139,140]. Of the reported chains of transmission that occurred in the West African EVD epidemic, all of the transmissions occurred after March 2015 which was well after the peak of the epidemic, suggesting that there were flare-up events [21,130,144]. A recent review by Subissi et al. provides a detailed analysis of the eight flare-ups that occurred at the end of the West African EVD epidemic, noting that all flare-ups have an association to a re-emergence event [138].

## 5. Conclusions

Although EBOV transmission was largely controlled towards the end of the West African epidemic, sporadic transmission events continued to occur. Asymptomatic EBOV testicular persistence, and subsequent sexual transmission, contributed to these events. Longitudinal studies of EVD survivors have demonstrated great variability in the duration of viral persistence. Alarmingly, EBOV genomic material has also been detected in semen from EVD survivors in the absence of physiological symptoms of disease following multiple prior negative samples. The development of EBOV vaccine candidates and novel therapeutics has resulted in mass vaccination campaigns during recent outbreaks and could hold promise for combating EVD in regions where the virus is endemic. However, although 36 clinical trials have been completed, only a single study has demonstrated clinical efficacy [145,146]. Thus, continued investigations of EVD clinical sequelae, including testicular persistence, are required to identify additional information that will aid both containment and patient management strategies.

Despite a growing number of reports describing the testicular persistence of EBOV, and additional filoviruses, in disease survivors, there is little information regarding the molecular mechanisms underlying these clinical sequelae. Currently, there is limited knowledge regarding the events that result in filovirus transmission to, and cell tropism within, the testis. Several critical questions remain, including:

(i) What cells are targeted by filoviruses in the testis?
(ii) What are the molecular mechanisms that contribute to filovirus testicular transmission?
(iii) How do viral fitness and host response modulation contribute to these asymptomatic EBOV testicular infections?
(iv) What are the kinetics of infectious virus and viral RNA shedding from sperm?
(v) What is the rate of EBOV sexual transmission?

These questions are of critical importance given the increasing frequency of filovirus outbreaks as they will help inform public health agencies to allow them to develop effective strategies to prevent human-to-human transmission.

## Figures and Tables

**Table 1:** Compilation of persistent filovirus infections in male survivors: 1967–2000.

Year of Detection	Location	Virus	Number Persistent Men	Days of Persistence											Reference
				0–90	91–180	181–270	271–360	361–450	451–540	541–630	631–720	721–810	811–900	901–990	
1967	Marburg, Germany	MARV	1		1										[100]
1976	Porton Down, UK	EBOV	1	1											[27,103]
1980	Nairobi, Kenya	MARV	1	1											[25]
1990	Siberia, Russia	MARV	1	1											[104]
1995-7	Kikwit, DRC ^1^	EBOV	4	2	2										[26]
1995-7	Kikwit, DRC ^1^	EBOV	4	3	1										[103]
2000	Gulu, Uganda	SUDV	1	1											[128]

^1^ Survivor and contact cohort studies.

**Table 2:** Compilation of male persistence cases reported during the West African EVD outbreak.

Year of Detection	Location	Number Persistent Men	Days of Persistence											Reference
			0–90	91–180	181–270	271–360	361–450	451–540	541–630	631–720	721–810	811–900	901–990	
2014-5	Dubreka, Guinea ^1^	1			1									[129]
2014-5	USA	5		3	1	1								[23]
2015	Kambia, Sierra Leone ^1^	1	1											[98]
2015	Monrovia, Liberia ^1^	1			1									[21,130]
2015	Guinea	8	3	2	2	1								[131]
2015	Bethesda, USA ^1^	1		1										[68]
2015-6	Guinea	19	6	8	4		1							[29]
2015-6	Guinea	15	4	4	2	2	1	1	1					[24]
2015-6	Liberia	38	2	2	6	4	16	6	2					[22]
2015-6	N’Zerekore, Guinea ^1^	1							1					[132]
2015-6	Sierra Leone	57	7	26	15	4	4	1						[62]
2016-7	Liberia	8							2	2	2	1	1	[133]

^1^ Cases included from case reports.

**Table 3:** Sexual transmission reports from 1967–2016.

Location	Acute Case Date	Transmission Date	Total Persistence (Mo.) ^1^	Transmission	Cases	Deaths	Virus	Reference
Marburg, Germany	8 November 1967	4 November 1967	4	Sex, Probable	1	0	MARV	[100]
Kikwit, Democratic Republic of Congo	Unknown	Unknown, 1995	Unknown	Sex, Inconclusive	1	0	EBOV	[103]
Monrovia, Liberia	20 March 2015	7 March 2015	6.5	Sex Confirmed	1	1	EBOV	[21,130]
Needowein, Liberia	29 June 2015	June 2015	10 ^2^	Sex, Possible	7	2	EBOV	[139]
Kambia, Sierra Leone	29 August 2015	August 2015	2	Sex, Confirmed	6	Unknown	EBOV	[98]
Conakry, Guinea	13 October 2015	September 2015	9 ^2^	Sex, Possible	2	0	EBOV	[140]
Bombali, Sierra Leone	12 September 2015	Unknown	Unknown	Sex, Possible	1	1	EBOV	[141,142]
Magburaka, Sierra Leone	14 January 2016	January 2016	13 ^2^	Sex, Possible	2	1	EBOV	[143]
N’Zerekore, Guinea	16 March 2016	20 February 2016	16	Sex, Confirmed	13	8	EBOV	[132]

^1^ Total reported length of persistence of the male survivor; ^2^ Probable cases with reported potential persistent survivor or matching sequence found, providing possible persistence lengths.
